# PD-L1 Expression in Different Segments and Histological Types of Ovarian Cancer According to Lymphocytic Infiltrate

**DOI:** 10.3390/medicina57121309

**Published:** 2021-11-29

**Authors:** Ljubiša Jovanović, Radmila Janković, Andja Ćirković, Milena Jović, Tijana Janjić, Slaviša Djuričić, Svetlana Milenković

**Affiliations:** 1 Department of Pathology and Medical Cytology, University Clinical Center of Serbia, 11000 Belgrade, Serbia; cecanaa63@gmail.com; 2 Faculty of Medicine, Institute of Pathology, University of Belgrade, 11000 Belgrade, Serbia; radmila.jankovic@med.bg.ac.rs; 3 Faculty of Medicine, Institute for Medical Statistics and Informatics, University of Belgrade, 11000 Belgrade, Serbia; andja.aleksic@gmail.com; 4 Institute of Pathology and Forensic Medicine, Military Medical Academy, 11000 Belgrade, Serbia; alhemastro@gmail.com; 5 Clinic for Gynecology and Obstetrics, University Clinical Center of Serbia, 11000 Belgrade, Serbia; tijana1002@gmail.com; 6 Department of Clinical Pathology, Mother and Child Health Care Institute of Serbia “Dr. Vukan Cupic”, 11070 Belgrade, Serbia; slavisa.djuricic@gmail.com; 7 Faculty of Medicine, University of Banja Luka, 78000 Banja Luka, Bosnia and Herzegovina

**Keywords:** ovarian cancer, PD-L1, lymphocytic infiltrate

## Abstract

*Background and Objectives*: Ovarian cancer is the leading cause of death among gynecological tumors. PD-1/PD-L1 immunoregulatory mechanism is activated in ovarian cancers. Lymphocyte infiltration is a significant factor that affects its expression. We analyzed the correlation between localization of lymphocytic infiltrate and PD-L1 expression in epithelial ovarian tumors. *Materials and Methods*: PD-L1 expression was analyzed in 328 subjects, 122 with epithelial ovarian carcinoma, 42 with atypical proliferative tumor, and 164 with benign epithelial ovarian tumor. Expression in central and invasive tumor parts in epithelial ovarian carcinoma was combined with the most pronounced lymphocyte reaction. Immunohistochemical analysis was performed using the tissue microarray and correlated with a set of histopathology parameters. *Results*: PD-L1 expression was most prominent in epithelial ovarian carcinoma with different levels of expression observed between invasive and central tumor segments. A high level of PD-L1 expression on tumor cells was more frequently present in the invasive than in the central tumor parts (*p* < 0.001) only in high-grade serous ovarian carcinoma (HGSC). There was no significant correlation between peritumoral lymphocytic infiltrate and PD-L1 expression regardless of tumor segment. In the central tumor parts of HGSC, there was a correlation of intratumoral lymphocytic infiltrate with a higher level of PD-L1 expression (*p* = 0.003). *Conclusions*: The most prominent PD-L1 expression was observed in the invasive tumor parts of HGSC. Only the central parts of the HGSC exhibited significant PD-L1 expression in association with considerable intratumoral lymphocytic infiltrate.

## 1. Introduction

Epithelial ovarian cancer (EOC) has the highest mortality rate among all gynecological tumors. It represents the eighth most common female cancer [1]. Due to the absence of early symptoms and screening programs, EOC is diagnosed mostly in advanced stages, with widespread metastasis and poor prognosis despite therapy. The lack of effective therapy and the frequent relapses indicates the need for new therapeutic strategies. Platinum-based chemotherapy is the main treatment for these patients. Despite new therapy, such as vascular endothelial growth factor (VEGF) and poly(ADP-ribose) polymerase (PARP) inhibitors, recurrences are still inevitable [2].

Suppression of the antitumor immune response is one of the crucial mechanisms by which tumor cells ensure their survival [3]. Tumors can suppress the host’s immune responses by activating physiological regulatory mechanisms (checkpoints) to escape immune detection. One such significant checkpoint is programmed death protein 1 (PD-1) and its ligand, programmed death-ligand 1 (PD-L1). PD-L1 expressed on tumor cells interacts with PD-1 receptor on T lymphocytes and leads to inhibition of secretion cytokines and deactivation of effector T lymphocytes. Such an interaction makes tumor cells resistant to effector lymphocytes [4,5]. The PD-1/PD-L1 pathway inhibition using monoclonal antibodies has become a promising therapy in many cancers with increased survival. Ovarian cancer cells can activate the PD-1/PD-L1 immunoregulatory mechanism and make tumors more aggressive, with poor therapy response and worse prognosis [6].

Tumor-infiltrating lymphocytes (TILs) contribute to reduced tumor growth [5]. The significance of TILs and their correlation with the prognostic parameters has been the subject of many studies [7,8]. In advanced carcinomas with less TIL, the antitumor immune response is weak [7]. Both intratumoral and peritumoral localizations of T lymphocytes are associated with better outcomes, with intratumoral localization being a slightly stronger prognostic parameter [8]. Accordingly, an association between prolonged survival in ovarian carcinoma patients and prominent TIL has been shown, regardless of histological type, grade, and stage of the tumor [9]. TIL expresses an effective memory immune response that contributes to long-term antitumor immunity. Effector T lymphocytes suppress potential recurrences [10]. Therefore, antitumor immunotherapy could be more effective in EOC with prominent TIL [7,8,10].

PD-L1 expression on ovarian cancer cells is induced by INF-γ from T lymphocytes in the tumor microenvironment [11]. Prominent TIL is associated with high PD-L1 expression on ovarian cancer cells in various histology types [12,13]. Expression levels of PD-L1 marker and TIL are independent prognostic parameters for patients with ovarian carcinoma [14,15]. There is an association between prominent TIL and PD-L1 expression on tumor cells, especially in the most invasive parts of the tumor. PD-L1 expression on ovarian tumor cells of high-grade serous carcinoma (HGSC) correlates negatively with patients’ survival, while prominent TIL contributes to better outcomes [16].

In this study, we analyzed the correlation between the localization of lymphocytic infiltrate and PD-L1 expression in epithelial ovarian tumors.

## 2. Materials and Methods

### 2.1. Study Population

The study included 328 subjects who underwent surgery due to epithelial ovarian tumor (EOT) in the period from 2017 to 2019 at the Clinic for Gynecology and Obstetrics, University Clinical Center of Serbia, Belgrade. The experimental group consisted of 122 subjects with epithelial ovarian carcinoma (EOC) and 42 with atypical proliferative tumor (ATP). The control group included 164 patients with benign epithelial ovarian tumor (BOT). The following parameters were recorded for each subject: patient’s age, menopausal status, histological type of tumor, tumor differentiation, International Federation of Gynecology and Obstetrics (FIGO) stage, presence of lymphovascular tumor invasion, necrosis, and intratumoral and peritumoral lymphocyte infiltration. Patients with secondary and non-epithelial ovarian tumors were excluded from the study. The ethical approval was obtained from the Ethics Committee of the University Clinical Center of Serbia. Written informed consent was obtained from all patients.

### 2.2. Tissue Microarray (TMA)

The tissue microarray (TMA) method was performed using two cylinders of tissue from each paraffin block with a 3 mm puncture needle. The first cylinder was taken from the central part of the tumor, and the second cylinder was taken from the peripheral part. Both parts of the tumor were with the most pronounced lymphocyte reaction. The cylinders were moved to a recipient paraffin block, where a series of 28 tissue cylinders was formed [17]. In the first row of each block, a placental tissue was placed to serve for orientation and as a positive internal control for immunohistochemical analysis [14].

### 2.3. Immunohistochemical Analysis

Immunohistochemical staining for PD-L1 was performed on TMA sections, on the Autostainer Link 48, Agilent, Denmark. For the PD-L1 antibody, epitope unmasking was done in EnVision FLEX epitope unmasking solution pH 6.1 (K8005, Agilent, Santa Clara, CA, USA). The visualization system EnVision FLEX (Agilent) was used for immunohistochemical analysis. A monoclonal anti-human PD-L1 antibody (clone 22C3, M3653, Agilent) in a dilution of 1:30 was used as the primary antibody. The analysis was performed on EOC samples (central and peripheral tumor parts), samples from ATP tumors, and samples from the BOT. Positive tumor cells were counted on the ×400 power field and their percentage from the total number of tumor cells was determined. Tumor cells usually show membrane staining for the PD-L1 antibody, with variable expression in the cytoplasm and nucleus. Any convincing partial or complete linear membrane PD-L1 staining in at least one viable tumor cell that is perceived as distinct from cytoplasmic staining we consider as a positive reaction [18,19]. The following score was used to describe the expression of PD-L1: negative (0) expression, without positive cells or with a single positive cell (<1%); low (1+) expression, with less than 10% positive cells; moderate (2+) expression, with 10–50% positive cells; and strong (3+) expression, with more than 50% positive cells. Tumors with moderate (2+) and strong (3+) positivity were considered as high PD-L1 expression.

### 2.4. Tumor-Infiltrating Lymphocytes (TILs)

**Figure 1:**
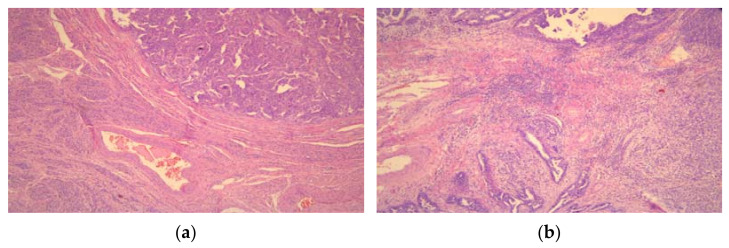
High-grade serous ovarian carcinoma (**a**) without and (**b**) with tumor-infiltrating lymphocytes (TILs) (×50).

The lymphocytic infiltrate was analyzed at whole slides before TMA constructions in the central and peripheral tumor parts and its localization was correlated with PD-L1 expression on tumor cells. The presence of lymphocytic infiltrate was estimated on microscopic magnification ×50 on HE tissue samples before microarray forming (Figure 1). Two categories were formed. Prominent lymphocytic infiltrate in EOCs was considered as TIL positive, while absent or rarely lymphocytes were designated as TIL negative [20].

### 2.5. Statistical Analysis

Statistical analyses were performed using Statistical Package for Social Sciences 20.0 (SPSS Inc., Chicago, IL, USA). Data were expressed as means ± standard deviation (SD) for continuous variables and percentages for categorical variables. Differences between groups for categorical data were tested by one-way ANOVA with Tuckey post hoc testing, Chi-square test or Fisher’s exact test. The degree of association between categorical data was calculated using the Mann–Whitney test. The differences between central and invasive tumor parts were done by the Chi-square test. Comparison of PD-L1 expression and lymphocytic infiltrate type was done by the Chi-square test or Fisher’s exact test. A *p*-value of less than 0.05 was considered statistically significant.

## 3. Results

### 3.1. Clinical and Histopathological Characteristics

The study included women with epithelial ovarian tumors classified into three study groups (Table 1). The mean age of all patients was 52.4 ± 15.8 years (age range, 15–84 years). Women with EOC were significantly older (*p* < 0.001) than women with ATP and BOT. The vast majority of women in the EOC group were menopausal (60.7%). The distribution of different histological types and FIGO stages is shown in Table 1.

The histopathological characteristics of EOCs are presented in Table 2.

### 3.2. Immunohistochemical Analysis of PD-L1 Expression

There was a statistically significant difference in the expression level of PD-L1 between EOC, ATP tumors, and BOT. Higher expression was proven in EOC than in ATP and BOT (Table 3).

**Figure 2:**
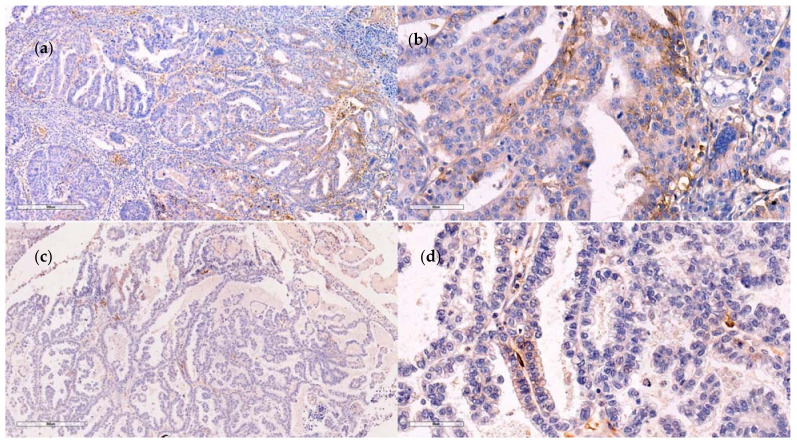
Different levels of PD-L1 expression in high-grade serous ovarian cancer cells. (**a**) (×100) and (**b**) (×400) strong PD-L1 expression, and (**c**) (×100) and (**d**) (×400) moderate PD-L1 expression.

The difference in intensity of PD-L1 expression in EOCs concerning patient and tumor characteristics is presented in Table 4. Bilateral EOCs had significantly more cases with a high intensity of PD-L1 expression (*p* < 0.001). The intensity of PD-L1 expression was higher in serous (79.6%) than in mucinous or endometrioid EOCs (*p* < 0.001). Additionally, it was higher in HGSC than in low-grade serous carcinoma (LGSC) (*p* = 0.007). A high level of PD-L1 expression was more frequent in EOCs with stage FIGO III and IV than in those with stage FIGO I and II (*p* < 0.001). A high level of PD-L1 expression was predominant in grade 3 EOCs (84.2%) (*p* < 0.001). Moreover, a high intensity of PD-L1 expression level was significantly more frequent in EOCs with tumor necrosis, lymphovascular invasion, and intratumoral and peritumoral infiltration. There was no difference in the intensity of PD-L1 expression when patients were stratified according to age groups, menopausal status, and tumor size. The examples of positive membranous PD-L1 staining in the most common high-grade serous ovarian cancer cells are shown in Figure 2.

### 3.3. PD-L1 Expression in Different Tumor Parts in Relation to the Localization of Lymphocytic Infiltrate

PD-L1 expression varied in frontal (invasive) and central tumor parts, but only in the HGSC was the statistically significant difference between these tumor parts shown (*p* < 0.001). A high level of PD-L1 expression was more frequently present in the invasive than in the central tumor parts (*p* < 0.001) (Table 5).

Intratumoral lymphocytic infiltrate was more frequently present (84.3%) in the central tumor parts, with a high level of PD-L1 expression (*p* = 0.003) (Table 6).

## 4. Discussion

PD-1/PD-L1 target therapy has recently attracted attention in EOC [2]. The efficiency of immunotherapy is based on the presence of target molecules that could be modulated by different therapeutic agents. PD-L1 receptor is one of the molecules whose functions are currently explored, especially concerning the other factors in the tumor microenvironment [14,20,21,22]. Immunohistochemical analysis of PD-L1 expression in tumor tissue is a prerequisite for the potential usage of PD-L1 inhibitors. Treatment outcomes should be better with this novel therapy than with standard chemotherapy agents alone [8,14,22]. Our study showed a significantly higher level of PD-L1 expression in EOCs than in ATP tumors or in controls, indicating that the PD-1/PD-L1 immunoregulatory mechanism is activated in EOCs, most frequently in HGSC. The distinction of PD-L1 expression between high- and low-grade serous carcinomas has been described previously, as well as its association with clinical outcome [23]. These findings underline the possibility for the usage of PD-L1 inhibitors in patients with more aggressive ovarian cancers, such as HGSC.

We noted a significant difference in PD-L1 expression between central and invasive tumor parts only in the HGSC type. Other histological types of EOC did not show a significant difference. Thus, a relatively small sample of non-HGSC tumors could be related to this statistical insignificance [18,19].

TILs are part of the endogenous immune reaction to the tumor cells, capable of recognition and elimination of the latter [22]. We found more pronounced TILs in HGSC with higher PD-L1 expression on tumor cells in comparison to other histological types. Higher PD-L1 expression in HGSC with more TILs can be explained as the upregulation of PD-L1 receptors on tumor cells by activated T lymphocytes [24]. Studies that analyzed the association between PD-L1 expression on tumor cells and the presence of TIL found a better prognosis in cases where carcinomas had more lymphocytic infiltrate [22,24,25,26].

We also demonstrated more frequent PD-L1 expression in HGSC with significant intratumoral lymphocytic infiltrate, especially in central tumor parts. Our assumption, however, that the invasive tumor parts with high PD-L1 expression correlate with the most prominent peritumoral lymphocyte infiltrate did not reach statistical significance. Some authors reported differences between peritumoral and intratumoral lymphocytes concerning PD-L1 expression in HGSC [8]. A positive correlation between intratumoral lymphocytic infiltrate and higher survival rates was found. Studies also reported a positive correlation between PD-L1 expression on tumor cells and favorable prognosis in HGSC [8,26]. Intraepithelial TILs were described as the most significant factor for the control of EOCs’ progression [22].

On the other hand, one study reported a negative correlation between PD-L1 expression on ovarian cancer cells and the presence of intratumoral lymphocytes [27]. The negative correlation is explained as inhibition of the antitumor immune response by PD-L1 molecules on cancer cells. Additionally, the small sample size and the methodological inconsistency (clone type) could be the factors contributing to these results. The same study defined PD-L1 expression and intratumoral lymphocytes as independent prognostic factors for EOCs [27].

One of the limitations of our study is that we did not analyze PD-L1 expression on immune cells in the tumor microenvironment. Studies of the distribution of PD-L1 and PD-1 expression on immune cells in HGSC and their diagnostic and prognostic significance found that there was a survival benefit in cases with high macrophage PD-L1 expression [28].

One recent study investigated PD-L1, PD-1, and CD8 expressions on TILs, and PD-L1 expression on ovarian cancer cells, and compared results in primary EOC with their expressions in peritoneal metastases [29]. The correlation was found only in the case of PD-L1 expression on tumor cells between primary EOC and peritoneal metastases. Marker expressions on TILs were significantly different in primary EOC compared to the peritoneal metastases. The higher PD-L1 expression on TILs was in correlation with unfavorable prognosis [29]. This observation could be explained by finding that PD-L1 on TILs can suppress neighboring T lymphocytes with PD-1 receptors and indicate intratumoral immune tolerance [30]. On the contrary, some researchers stated that a high level of PD-L1 expression on TILs was a protective factor for EOC. TILs with PD-L1 molecules on the surface could increase the level of cytotoxic CD8+ T lymphocytes and therefore promote antitumor response [21]. Additional investigation of PD-L1 status on TILs, especially in association with PD-1 expression, is an important subject for further research and provision of a better understanding of the immune antitumor regulation in EOC.

Regardless of recently involved drugs in EOC treatment as Bevacizumab and Olaparib, the prognosis for these patients is still poor. Implementation of PD-L1 inhibitors should be a promising strategy in EOC treatment. However, previous studies described the weak response by PD-L1 inhibitors for these patients. Different components of the EOC microenvironment contribute to this failure [31]. Significant heterogeneity in EOC could be an additional factor for this inadequate response. In HGSC at least four genomic classes were identified differing for immunoreactivity [31,32]. Therefore, a comprehensive analysis of the genomic status of EOC and immunological characteristics in the tumor microenvironment could be predictive factors for immune therapy response [2,32]. We believe that additional analysis of interactive signaling pathways could improve strengthen of PD-L1 inhibitors. Lymphocyte activation gene (LAG-3) and a cluster of differentiation 27 (CD27) are such molecules with the immunomodulatory role that are worth further researching [32].

Recent studies have shown insufficiently reliable PD-L1 staining in EOC for the usage of PD-L1 inhibitors. Some patients without PD-L1 expression had a satisfying therapy response to PD-L1 inhibitors. On the contrary, there were patients without therapy effects despite positive PD-L1 expression. The explanation could be tumor heterogeneity, which is quite pronounced in metastatic tumor sites. It leads to different PD-L1 values suggesting immunohistochemistry analysis in both primary and metastatic tumors for the increased prognostic potential of PD-L1 marker [33].

Many trials of usage of PD-L1 inhibitors in EOC have shown limited success. Patient selection based on simultaneous PD-L1 analysis in cancer cells and evaluation of TILs could improve these results [11]. One study reported about increased efficacy of immune therapies in HGSC if they activate adequate intercellular pathways in the tumor microenvironment, including TIL [34].

Studies reported a positive correlation between TILs, PD-L1 expression on ovarian cancer cells, and the presence of breast cancer genes 1 and 2 (BRCA1 and 2) mutations. The association between these parameters could imply the potential for the usage of immune therapy in ovarian cancers with BRCA mutations [25]. The synergistic use of PARP inhibitors for these patients could be promising therapeutic strategies in the future to enhance the clinical effectiveness of immunotherapy [25,32]. PARP inhibitors induce the release of neoantigens and enhance PD-L1 expression [32]. Mismatch repair (MMR) deficiency is also represented as a mechanism of immune responsiveness [32]. Previous studies reported about the association between PD-L1 expression and microsatellite instability status [33].

Overall survival (OS) is worse in many cancers with high PD-L1 expression. Some studies represented the inconsistent OS outcomes of PD-L1 expression in ovarian HGSC. It could be because of various detection and scoring systems and differences in sample size. The presence of antitumor immune response as TILs predicts significantly better OS in EOC. Correlation between prolonged OS and the presence of TILs indicates that EOCs are for certain intrinsically immunogenic tumors [5].

PD-L1 expression in HGSC was approached in only a few studies with variable results [5]. There is a lot of contradiction about the PD-L1 expression on tumor cells concerning the type of lymphocytic infiltrate. In this study, we additionally analyze expression status in different tumor parts and among different histological types of EOC. Our study reports about the significance of the tumor microenvironment and antitumoral immune response, which is more expressed in advanced FIGO stages and more aggressive histological EOC types (HGSC). This study involved a large number of EOTs with different biological behaviors and histological types, which should improve the validity of the obtained results.

The analysis of PD-L1 expression with assessment TIL status could become a promising therapeutic target for patients with ovarian HGSC [5]. We need more studies to validate their therapeutic potential. The analysis of a combination of different therapy modalities could bring to better outcomes and longer OS for patients with HGSC [5].

## 5. Conclusions

We confirmed higher PD-L1 expression in an aggressive histologic type of EOC (HGSC) in advanced FIGO stages. Invasive tumor parts of HGSC showed the most frequent PD-L1 expression, but only in the central parts of HGSC there was significant PD-L1 expression associated with remarkable intratumoral lymphocytic infiltrate. Our study results support the hypothesis that the PD-L1 inhibitors could be an effective therapeutic option in aggressive ovarian carcinomas as HGSC, especially with prominent intratumoral lymphocytic infiltrate.

## Figures and Tables

**Table 1:** Demographic, clinical characteristics, and histopathological types of tumors.

	Total *n* = 328	EOT			*p*	EOC vs. ATP	EOC vs. BOT	ATP vs. BOT
		EOC *n* = 122	ATP *n* = 42	BOT *n* = 164				
Age, mean ± sd (years)	52.4 ± 15.8	61.8 ± 10.1	45.8 ± 12.2	47.2 ± 15.6	<0.001	<0.001	<0.001	0.834
Menopause, *n* (%)								
Yes	199 (60.7)	106 (86.9)	20 (47.6)	73 (44.5)	<0.001	<0.001	<0.001	0.718
No	129 (39.3)	16 (13.1)	22 (52.4)	91 (55.5)				
Histological type, *n* (%)								
Serous	206 (62.8)	103 (84.4)	25 (59.5)	78 (47.6)	<0.001	<0.001	<0.001	0.044
Mucinous	112 (34.1)	10 (8.2)	16 (38.1)	86 (52.4)				
Endometrioid	10 (3.0)	9 (7.4)	1 (2.4)	0 (0.0)				
FIGO stage, *n* (%)								
I + II	81 (49.4)	39 (32.0)	42 (100.0)	/	<0.001	<0.001	NA	NA
III + IV	83 (50.6)	83 (68.0)	0 (0.0)	/				

EOT—epithelial ovarian tumor, EOC—epithelial ovarian carcinoma, ATP—atypical proliferative tumor, BOT—benign ovarian tumor, FIGO—International Federation of Gynecological and Obstetrics.

**Table 2:** Histopathological characteristics of EOCs.

Characteristic	EOCs *n* = 122			*p* ^a^	*p* ^b^	*p* ^c^
	Serous *n* = 103	Mucinous *n* = 10	Endometrioid *n* = 9			
Differentiation (Grade), *n* (%)						
Well and moderately differentiated (Grade 1 + Grade 2)	12 (11.7)	9 (90.0)	6 (66.7)	<0.001	<0.001	0.213
Poorly differentiated (Grade 3)	91 (88.3)	1 (10.0)	3 (33.3)			
Lymphovascular invasion, *n* (%)	95 (92.2)	4 (40.0)	4 (44.4)	<0.001	<0.001	1.000
Necrosis, *n* (%)	88 (85.4)	5 (50.0)	4 (44.4)	0.005	0.001	1.000
Intratumoral lymphocyte infiltration, *n* (%)	89 (86.4)	3 (30.0)	7 (77.8)	<0.001	<0.001	0.070
Peritumoral lymphocyte infiltration, *n* (%)	96 (93.2)	6 (60.0)	8 (88.9)	0.008	0.003	0.303

^a^ Serous vs. Mucinous, ^b^ Serous vs. Endometrioid, ^c^ Mucinous vs. Endometrioid. EOC—epithelial ovarian carcinoma.

**Table 3:** PD-L1 expression levels in epithelial ovarian tumors.

PD-L1 Expression Level	Group			*p*	EOC vs. ATP	EOC vs. BOT	ATP vs. BOT
	EOC	ATP	BOT				
0	0 (0.0)	7 (16.7)	159 (97.0)	<0.001	<0.001	<0.001	<0.001
1+	34 (27.9)	30 (71.4)	5 (3.0)				
2+	86 (70.5)	5 (11.9)	0 (0.0)				
3+	2 (1.6)	0 (0.0)	0 (0.0)				

EOC—epithelial ovarian carcinoma, ATP—atypical proliferative tumor. BOT—benign ovarian tumor.

**Table 4:** PD-L1 expression in relation to clinical and histopathologic characteristics of EOCs.

Characteristic	Categories		PD-L1 Expression				*p*
			Absent/Low *		High *		
Age	<65		21 (31.8)		45 (68.2)		0.291
	65+		13 (23.2)		43 (76.8)		
Menopausal status	Yes		28 (26.4)		78 (73.6)		0.357
	No		6 (37.5)		10 (62.5)		
Localization	Unilateral		20 (50.0)		20 (50.0)		<0.001
	Bilateral		14 (17.1)		68 (82.9)		
EOC size (mm)	<80		14 (24.6)		43 (75.4)		0.445
	≥80		20 (30.8)		45 (69.2)		
Histological type	Serous	HGSC	21 (20.4)	15 (16.5)	82 (79.6)	76 (83.5)	<0.001
		LGSC		6 (50.0)		6 (50.0)	
	Mucinous		7 (70.0)		3 (30.0)		<0.001
	Endometrioid		6 (66.7)		3 (33.3)		
FIGO stage	I + II		30 (76.9)		9 (23.1)		<0.001
	III + IV		4 (4.8)		79 (95.2)		
Differentiation (Grade)	1		15 (100.0)		0		<0.001
	2		4 (33.3)		8 (66.7)		
	3		15 (15.8)		80 (84.2)		
Necrosis	Yes		19 (19.6)		78 (80.4)		<0.001
	No		15 (60.0)		10 (40.0)		
Lymphovascular invasion	Yes		18 (17.5)		85 (82.5)		<0.001
	No		16 (84.2)		3 (15.8)		
Intratumoral lymphocyte infiltration	Yes		20 (20.2)		79 (79.8)		<0.001
	No		14 (60.9)		9 (39.1)		
Peritumoral lymphocyte infiltration	Yes		27 (24.5)		83 (75.5)		0.013
	No		7 (58.3)		5 (41.7)		

EOC—epithelial ovarian carcinoma, FIGO—International Federation of Gynecological and Obstetrics. * absent/low PD-L1 expression (0 and 1+), high PD-L1 expression (2+ and 3+).

**Table 5:** PD-L1 expression in the different parts of EOC: in the center and the invasive front.

Categories of PD-L1 Expression	Tumor Localization		*p*
	Center	Invasive Front	
HGSC			
1+	15 (16.5)	3 (3.3)	<0.001
2+	74 (81.3)	60 (65.9)	
3+	2 (2.2)	28 (30.8)	
LGSC			
1+	6 (50.0)	1 (8.3)	0.069
High expression (2+ and 3+)	6 (50.0)	11 (91.7)	
Mucinous EOC			
1+	7 (70.0)	5 (50.0)	0.650
2+	3 (30.0)	5 (50.0)	
Endometrioid EOC			
1+	6 (66.7)	3 (33.3)	0.347
2+	3 (33.3)	6 (66.7)	

EOC—epithelial ovarian carcinoma, HGSC—high-grade serous carcinoma, LGSC—low-grade serous carcinoma.

**Table 6:** PD-L1 expression categories of HGSC according to the presence of intratumoral and peritumoral lymphocytic infiltrate.

Tumor Part	Categories of PD-L1 Expression	Intratumoral Lymphocytic Infiltrate		*p*	Peritumoral Lymphocytic Infiltrate		*p*
		with	without		with	without	
Center	absent/low *	14 (15.7)	7 (50.0)	0.003	18 (18.8)	3 (42.9)	0.126
	High **	75 (84.3)	7 (50.0)		78 (81.3)	4 (57.1)	
Invasive front	absent/low *	2 (2.2)	2 (14.3)	0.088	3 (3.1)	1 (14.3)	0.249
	high *	87 (97.8)	12 (85.7)		93 (96.9)	6 (85.7)	

* absent/low PD-L1 expression (0 and 1+), ** high PD-L1 expression (2+ and 3+).

## Data Availability

Data available in a publicly accessible repository.
